# PERCEPTION Trial protocol: Comparison of predictive and prognostic capacities of neutrophil, lymphocyte, and platelet counts and tumor-infiltrating lymphocytes in triple negative breast cancer

**DOI:** 10.1097/MD.0000000000023418

**Published:** 2020-12-11

**Authors:** Sejdi Lusho, Xavier Durando, Yannick Bidet, Ioana Molnar, Myriam Kossai, Maureen Bernadach, Nathalie Lacrampe, Hugo Veyssiere, Mathias Cavaille, Mathilde Gay-Bellile, Nina Radosevic-Robin, Catherine Abrial

**Affiliations:** a Université Clermont Auvergne, INSERM UMR 1240 «Imagerie Moléculaire et Stratégies Théranostiques», Centre Jean Perrin, 58 rue Montalembert, 63000 Clermont-Ferrand; b Délégation Recherche Clinique & Innovation, Centre Jean Perrin, 58 rue Montalembert, F-63011 Clermont-Ferrand; c Centre d’Investigation Clinique, UMR501, F-63001 Clermont-Ferrand; d Centre Jean PERRIN, Laboratoire d’oncologie moléculaire; e Centre Jean Perrin, Département d’anatomie et de cytologie pathologiques; f Centre Jean Perrin, Département d’oncogénétique, 58 rue Montalembert, 63011 Clermont-Ferrand, France.

**Keywords:** full blood count, metastatic recurrence, neutrophil-to-lymphocyte ratio, predictive factors, study protocol, triple negative breast cancer, tumor-infiltrating lymphocytes

## Abstract

**Background:**

Triple negative breast cancer affects 10% to 20% of all women diagnosed with breast cancer. Due to its characteristics, treatment strategies are limited and metastatic recurrences are common in the first 5 years after treatment. However, not all patients affected by this disease develop metastases. Tumor-infiltrating lymphocytes have shown to be reliable predictive biomarkers of treatment response and metastatic recurrences. However, we need to develop simpler and faster ways to predict response to cytotoxic treatment and the possibility of eventual cancer relapse by identifying new biomarkers. Recently, new studies are emerging, suggesting a predictive role of circulating blood cells in different types of cancer. In this study, we will assess the correlation between tumor-infiltrating lymphocytes and different elements of the blood count in patients diagnosed with triple negative breast cancer.

**Methods:**

The main objective of this study is to evaluate the correlation between the peripheral neutrophil-to-lymphocyte ratio and the amount of tumor-infiltrating lymphocytes, assessed in triple negative breast cancer patients at diagnosis. Secondary objectives include evaluation of the correlation between tumor-infiltrating lymphocytes at diagnosis and the baseline absolute neutrophil, lymphocyte, and platelet counts, as well as the platelet-to-lymphocyte ratio. The triple negative breast cancer patients will be enrolled in the PERCEPTION trial during the first year after the treatment completion. Two supplementary blood tests, at 12 months after the end of treatment and at the time of the first metastatic recurrence, will be performed.

**Discussion:**

The discovery of new prognostic and predictive biomarkers is crucial for triple negative breast cancer. We set up the PERCEPTION clinical trial in order to evaluate certain blood counts as early biomarkers and to assess their correlation with tumor-infiltrating lymphocytes. Demonstration of comparative predictive and/or prognostic capacities of peripheral blood counts and tumor-infiltrating lymphocytes would allow introduction of the former as simple and cheap biomarkers in triple negative breast cancer patient management.

**Trial registration:**

The PERCEPTION study has been registered in the French National Agency of Medical Security registry on the 2nd of July 2019 under the number 2019-A01861-56 and in the ClinicalTrials.org registry under the number NCT04068623.

## 1 Introduction

Triple negative breast cancer (TNBC) represents 10% to 20% of all breast cancers. Treatment strategies for this type of breast cancer are limited, due to the lack of hormone receptor expression (oestrogen receptors and progesterone receptors) and the absence of amplification for the gene coding for the Human Epidermal Growth Factor Receptor 2 (HER2) protein, in tumor cells.^[1]^ As a result, patients diagnosed with TNBC cannot benefit from endocrine therapy or anti-HER2 therapy. Therefore, TNBC has an unfavorable prognosis with a high rate of metastatic recurrence during the first 5 years after treatment.^[2–4]^ However, not all TNBC patients develop metastatic recurrence and it is therefore crucial to identify biomarkers that would allow determining which patients will eventually relapse.

Several studies have demonstrated that higher amounts of tumor-infiltrating lymphocytes (TILs) in the biopsy before treatment are associated with higher rate of pathologic complete response (pCR) to the neoadjuvant chemotherapy (NACT).^[5]^ Other studies have shown a correlation between the presence of high TIL quantity in the surgical specimen after NACT and a better relapse-free survival, as well as a significantly reduced death rate, in patients suffering from TNBC.^[6,7]^ For instance, Dieci et al have found that the 5-year overall survival rate for patients with high quantities of TILs in the neoadjuvant chemotherapy setting was 91% and significantly higher than that of patients with low quantities of TILs whose 5-year overall survival rate was 55%.^[6]^

The assessment of TIL quantity in a cancer tissue specimen is a semi-quantitative method, which is still not in routine use due to the need for a highly skilled pathologist to perform it, and, also, due to certain level of the inherent error risk. Therefore, reliable quantitative biomarkers that are easy to measure and that could provide additional information to that of TILs or even replace them in predicting treatment response and disease relapse are still lacking.

Various blood cell counts and the ratios between them, such as neutrophil-to-lymphocyte ratio (NLR) and platelet-to-lymphocyte ratio (PLR) could be used as predictive biomarkers in several types of cancer, including breast cancer.^[1,8,9]^ The research conducted by Lee et al in 2019 suggests that low NLR at baseline is associated with a better overall survival in TNBC patients.^[1]^

Besides the full blood count, several studies have shown a correlation between the presence of circulating tumor DNA (ctDNA) in the blood and treatment response or even metastatic recurrence.^[10,11]^ Despite the insufficient data in TNBC, studies have shown that ctDNA is a reliable marker in predicting cancer recurrence, due to its high sensitivity and specificity.^[12,13]^ Moreover, this circulating biomarker can be obtained and quantified from a simple blood test. Therefore, evaluation of ctDNA as a predictor of metastatic relapse in TNBC is of great interest and it could provide additional information to that of TILs and/or the circulating blood cell counts.

## 2 Patients and methods

### 2.1 Study aim

The aim of this study is to evaluate the correlation between the absolute counts of blood neutrophils, lymphocytes, and platelets, as well as the NLR and PLR, and the amount of TILs assessed on hematoxylin and eosin (H&E)-stained breast tumor tissue sampled before treatment for women with TNBC. Evaluation of this correlation might provide important information whether peripheral blood counts and their ratios could replace TILs in the prediction of TNBC treatment response and metastatic recurrence. Another aim of this study is to assess ctDNA as a biomarker of metastatic cancer recurrence.

### 2.2 Primary objective and endpoints

The primary objective of the PERCEPTION study is to evaluate the correlation between the amount of TILs found in the biopsy, measured semi-quantitatively as the percentage of tumor stromal area occupied by lymphocytes^[14]^ and the NLR derived from the absolute blood counts of neutrophils and lymphocytes in TNBC patients, at the time of diagnosis. The primary endpoints are the value of NLR and the amount of TILs at diagnosis (before any treatment). NLR is defined as the ratio of the absolute neutrophil count (ANC) over the absolute lymphocyte count (ALC) (NLR = ANC/ALC).

### 2.3 Secondary objectives and endpoints

Secondary objectives of the present study include:

- Assessment of the correlation between the amount of TILs in the biopsy and the total white blood cell count (WBC), ANC, ALC, absolute platelet count (APC), and PLR routinely assessed in women with TNBC, at diagnosis.
- Assessment of the correlation between the mentioned blood counts and their ratios (NLR and PLR), at the time of diagnosis, and the response to NACT according to Sataloff.^[15]^
- Assessment of the correlation between the mentioned blood counts and their ratios (NLR and PLR) at the time of diagnosis, and the TIL amount in the post-NACT surgical specimen.
- Assessment of the correlation between the mentioned blood counts and their ratios (NLR and PLR) and the recurrence-free survival (RFS).
- Assessment of the correlation between the mentioned blood counts and their ratios (NLR and PLR) before post-NACT surgery and TIL quantity in the surgical specimen post-NACT, depending on the time between surgery and the last administration of chemotherapy.
- Assessment of the correlation between the mentioned blood counts and their ratios (NLR and PLR) and the presence of ctDNA.
- Assessment of the correlation between ctDNA and RFS.
- Assessment of the correlation between the mentioned blood counts and their ratios (NLR and PLR) after cancer treatment and the RFS.

The secondary endpoints include the following:

- The absolute values of total white blood cells, neutrophils, lymphocytes, platelets measured in number of cells × 10^12^/L and the PLR at diagnosis. PLR is defined as the ratio of the APC over the ALC (PLR = APC/ALC).
- The values of total white blood cells, neutrophils, lymphocytes, platelets, NLR, and PLR before post-NACT surgery.
- TIL amount in the biopsy before cancer treatment.
- TIL amount in the surgical specimen post-NACT.
- Response to NACT according to the Sataloff classification.
- The period of time between surgery and the last administration of chemotherapy.
- The amount of ctDNA.
- Metastatic recurrence.

### 2.4 Eligibility criteria

The participants should meet the inclusion criteria in order to be enrolled in the study (see Table 1). Only adult (over 18 years of age) female patients diagnosed for the first time with non-metastatic TNBC will be recruited. The eligible patients must be treated with chemotherapy, surgery, and radiation therapy (RT). The lack of tumor sample from biopsy, necessary for the evaluation of TILs and the lack of blood test results at diagnosis will render the inclusion impossible.

**Table 1:** Inclusion and exclusion criteria of the PERCEPTION trial.

Inclusion criteria	Exclusion criteria
Female	Unavailable biopsy tumor samples
Age ≥18 yr	Unavailable blood test results at the time of diagnosis
Primitive, non-metastatic triple negative breast cancer, diagnosed histologically	Pregnant or breastfeeding women, vulnerable persons, adults under legal protection, or unable to give their consent, according to the L.1121-6 article of the Public Health Code
Treated with chemotherapy, surgery, and radiation therapy	
French language fluency	
Affiliation to social security	
Able to provide a signed informed consent form	

Patient may exit study prematurely following:

- Patient decision
- Investigator decision
- Patient death
- Patient lost to follow-up

### 2.5 Study design

**Figure 1:**
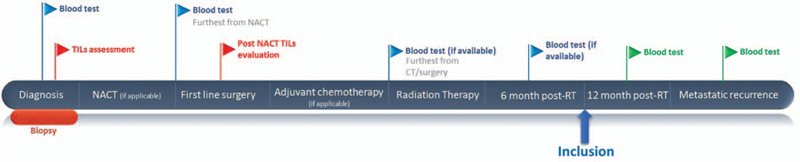
Timeline of the patients’ treatment and follow-up. CT = chemotherapy, NACT = neoadjuvant chemotherapy, RT = radiation therapy. Patients are included in the trial 1 year after the end of radiation therapy, as indicated by the blue arrow.

Eligible patients will be selected out of a local database of TNBC patients and will be recruited during the first year following the end of RT. The data collected before inclusion, such as TILs evaluation at baseline and in the surgical specimen, as well as blood test results at different times (baseline, before surgery, before the start of RT, and 6 months after the end of RT) will also be analyzed. After inclusion in the PERCEPTION trial, patients will undergo 2 interventions. A blood test will be performed 12 months after the end of RT and another blood test will be performed at the time of the first metastatic recurrence, if applicable. WBC, ANC, ALC, APC, NLR, PLR, and ctDNA will be measured from these blood tests. The overall follow-up is 5 years (see Fig. 1).

### 2.6 Recruitment

Recruitment will be carried out in the Jean Perrin Centre by an oncologist. After checking the inclusion and exclusion criteria, the study will be thoroughly explained to the patient by the oncologist, and a consent form will be signed by both.

The recruitment period will last 3 years, from October 2019 to October 2022; 90 patients are expected to be included. After enrolment completion, the primary objective will be analyzed. The overall follow-up for each patient is 5 years, if no metastatic recurrences occur.

### 2.7 Data collection and management

Most of the data will be available and will have been collected at the time of enrolment:

- Demographic data (age)
- Initial diagnosis of TNBC (date of diagnosis, histological characteristics of the tumor, hormone receptor and HER2 expression, TNM classification)
- Cancer treatment and response to the treatment
- Clinical characteristics of the tumor in the surgical specimen
- Blood test results at the time of diagnosis, before surgery, before the onset of RT, and 6 months post-RT
- TIL amount in the biopsy and in the surgical specimen

After patient enrolment, blood test results at 12 months post-RT and at the time of the first metastatic recurrence will be collected.

A comparison of the demographic and recurrence data of the recruited patients to those of eligible patients but not included in the study will be performed, to show that the studied population in this trial is no different from the general population of non-metastatic TNBC patients.

The assessment of TIL quantity will be independently performed by 2 breast pathologists. In case of the initial result discordance, the slides will be reviewed by both pathologists under a multi-headed microscope and the consensus will be reached after a concertation.

Tumor DNA mutations will be identified from tumor samples. Then, DNA will be extracted from the blood plasma and will be amplified by polymerase chain reaction (PCR), in order to select the DNA region carrying the mutation identified in the tumor sample and to measure the quantity of circulating tumor DNA.

Data will be collected and entered in the electronic Case Report Form (eCRF) by a Clinical Research Associate (CRA) in charge of the study, under the responsibility of the principal investigator.

### 2.8 Data confidentiality

Data collected and transmitted to the promoter by the investigators will be anonymized. Study data will not contain any names or other personal identifiers such as addresses. Patients included in the trial will be identified by a code specific to this trial. The investigator will have access to the correspondence table between the patient's last name, first name, date of birth, and the code assigned in the trial.

### 2.9 Statistical analysis

Given the scarcity of data in the literature concerning the correlation between TILs and NLR, our goal is to recruit as many patients as possible. A sample size of 90 patients would allow us to show an average effect size of 0.3 (with a 0.8 power and a 0.05 alpha risk). According to preliminary studies (not published), the variables present enough variability to allow for the detection of a correlation.

Statistical analysis will be performed with R software (v3.6 or newer, R-Project, GNU GPL, http://cran.r-project.org/). Each variable of interest will be expressed by conventional statistical summaries: the quantitative variables will be expressed through the most relevant position indicator (mean, median, quartiles, range) and dispersion indicators (standard deviation, interquartile range), and qualitative variables will be expressed by counts and percentages for each level.

Normality of continuous variables will be investigated and we will perform a description of the dependencies of the pairs of variables, where relevant. For hypothesis testing, alpha risk will be set at the conventional 5%, *P*-value corrections will be made for multiple comparisons, and effect sizes will be systematically specified. If relevant, post-hoc tests and comparisons in subgroups will also be performed for exploratory purposes and with adjustment for multiple testings.

### 2.10 Main objective analysis

In order to study the dependency relationship between NLR and TILs, we will perform a correlation analysis between these 2 continuous variables (graphical analysis, Spearman correlation coefficient, significance test, confidence interval).

### 2.11 Secondary objective analysis

- The relationship between the other blood counts and the TILs at different times will be assessed through a correlation analysis as for the main objective.
- The relationship between the absolute blood counts and the derived ratios (NLR, PLR), and the response to NACT will be evaluated by analyses of variance (or Kruskal–Wallis tests) and logistic regressions. A subgroup analysis will be performed based on the length of the period between the last cycle of NACT and surgery. A mixed model will also be considered to account for repeated measures and the time lag between the end of NACT and surgery.
- The relationship between the presence of ctDNA after the end of treatment and the relapse of TNBC will be analyzed by Fisher's exact test. Patients treated with NACT and first-line surgery will be analyzed separately.
- The elements of blood will be assessed as early markers of TNBC relapse using logistic regressions, possibly penalized (the LASSO method). Patients treated with NACT and first-line surgery will be analyzed separately.

## 3 Quality control

A CRA mandated by the promoter will ensure the proper conduct of the study, data collection, documentation, recording, and report, in accordance with the good clinical practices guidelines and the legal provisions in force.

The respect of the study protocol and procedures therein, and the quality of the collected data (accuracy, missing data, consistency with the source documents) will be regularly reviewed; monitoring reports will ensure traceability.

## 4 Amendment to the protocol

Changes to the protocol will be qualified as substantial or not. Depending on their nature, they will be the subject of a new review by the French ethical research committee and/or an authorization from the competent authority.

## 5 Discussion

Identifying new biomarkers that would allow for a quick, inexpensive, and reliable prediction of treatment response and metastatic recurrence of TNBC patients is still an important unsatisfied medical need. Such biomarkers would allow oncologists to propose alternative treatment and enrolment into clinical trials to the TNBC patients carrying a high risk of resistance to the classical NACT or/and a high risk of metastatic recurrence.

The PERCEPTION clinical trial is designed to verify whether a peripheral blood count or a ratio of peripheral blood counts can provide similar prognostic information as TILs. However, this clinical trial has certain drawbacks. First of all, since it is conducted at a single site, the sample size will be relatively limited. Furthermore, this study will only assess the full blood count and ctDNA as predictors of relapse. Studying other elements, such as circulating proteins would be of great interest in the prediction of TNBC relapse.

In conclusion, the PERCEPTION trial will give us important information about the correlation between NLR and TILs before treatment in TNBC patients and at other time periods. It will allow us to determine if we can further explore the values of blood cells as predictive markers in TNBC, and propose further studies, assessing new biomarkers that could be of great value in the treatment of TNBC.

## 6 Trial status

As of this day 18 patients have been recruited in the PERCEPTION trial. Recruitment began on 4th of December 2019 and is ongoing, according to version 14 of the protocol of 9th October 2019. Recruitment is expected to be completed in October 2022.

## Author contributions

NRR and XD conceived the study and contributed equally to this trial. NRR and SL performed the literature research. All authors designed the study. SL wrote the first draft of the article. CA, NRR, and XD revised the article. All authors read and approved the final manuscript. TILs evaluation will be carried out by MK and NL. Collection of data will be performed by SL. IM will be responsible for the data analysis.

**Conceptualization:** Xavier Durando, Yannick Bidet, Maureen Bernadach, Mathias Cavaille, Mathilde Gay-Bellile, Nina Radosevic-Robin, Catherine Abrial.

**Funding acquisition:** Sejdi Lusho, Xavier Durando.

**Investigation:** Xavier Durando, Myriam Kossai, Maureen Bernadach, Nathalie Lacrampe, Mathias Cavaille, Mathilde Gay-Bellile, Nina Radosevic-Robin.

**Methodology:** Sejdi Lusho, Xavier Durando, Ioana Molnar, Maureen Bernadach, Nina Radosevic-Robin, Catherine Abrial.

**Project administration:** Sejdi Lusho, Hugo Veyssiere.

**Software:** Ioana Molnar.

**Supervision:** Xavier Durando.

**Visualization:** Sejdi Lusho.

**Writing – original draft:** Sejdi Lusho.

**Writing – review & editing:** Xavier Durando, Ioana Molnar, Nina Radosevic-Robin, Catherine Abrial.
